# *Mussaenda saxatilis* (Rubiaceae), a new limestone species from Guangxi, China

**DOI:** 10.3897/phytokeys.280.207010

**Published:** 2026-10-14

**Authors:** Jin Li, Xiao-Yu Wei, Ting-Ting Duan

**Affiliations:** 1 Guangdong Ocean University, Zhanjiang CN-524088, Guangdong, China Guangdong Ocean University Zhanjiang China

**Keywords:** Karst flora, new species, Rubiaceae, southern China, taxonomy

## Abstract

A new species of *Mussaenda* (Rubiaceae), *M. saxatilis*, is described from the limestone karst regions of Guangxi, southern China. Morphologically, the new species most closely resembles *M. hainanensis* but differs from that species in having shallowly or moderately 2-lobed stipules, subsessile inflorescences, rotundate-ovate corolla lobes, smaller berries, and a pericarp that lacks lenticels. A detailed description, habitat information, phenology, distribution, and comparisons with morphologically similar species are provided.

## Introduction

*Mussaenda* L. is the largest genus in Mussaendeae (Dialypetalanthoideae, Rubiaceae), encompassing ca. 135 species worldwide, with ca. 30 species recorded in China (Chen and Taylor 2011; Razafimandimbison and Rydin 2024). It is distributed in Africa and Asia, with Asia representing the center of its diversity (Alejandro et al. 2005; Deng 2007; Duan et al. 2018). Species in this genus are small trees, shrubs, and twining shrubs typically growing at the edges of secondary forests, easily distinguished by their enlarged, petaloid calycophylls (Alejandro et al. 2005; Chen and Taylor 2011; Duan et al. 2018). Several species are widely cultivated as ornamental plants for horticulture (e.g., *M. erythrophylla* Schumach. & Thonn., *M. frondosa* L., *M. philippica* A.Rich., and *M. elegans* Schumach. & Thonn.) or as traditional herbal medicine (*M. pubescens* W.T.Aiton, *M. erosa* Champ. ex Benth.) (Gong et al. 2015; Zhang et al. 2024). Over the past two decades, increasing taxonomic attention has resulted in the discovery of numerous additional *Mussaenda* species in Asia (Alejandro et al. 2008; Deng and Zhang 2008; Duan et al. 2016, 2019). Recent taxonomic studies have considerably improved the understanding of species diversity within *Mussaenda*, particularly in tropical Asia (Alejandro et al. 2005; Deng 2007; Duan et al. 2018; Razafimandimbison and Rydin 2024).

During a long-term taxonomic revision of *Mussaenda*, extensive numbers of herbarium specimens were examined. While sorting specimens collected from Guangxi, several individuals morphologically distinguishable from all previously known species were discovered. These populations are morphologically similar to *M. hainanensis* Merr. (some specimens were previously identified as this species) but differ from the latter by their distally bifid stipules, lanceolate to narrowly elliptic calyx lobes, and fruits lacking lenticels. Following detailed field investigations of flowering and fruiting plants during several field surveys, the unusual plant was confirmed as a new species of *Mussaenda*, which is described below.

## Materials and methods

Morphological characteristics were studied through field observations of living plants conducted from August 2024 to May 2026, combined with examination of herbarium specimens at IBSC, IBK, GXMI, and PE. Field-collected specimens were measured manually with rulers, whereas digitized specimen data from online herbaria were quantified using Fiji (ImageJ v. 1.54j). To identify morphological differences from related species, specimens from the herbaria HUH, NY, IBSC, IBK, GXMI, PE, KUN, and NAS were examined through online databases, including JSTOR Global Plants (https://plants.jstor.org/), CVH (https://www.cvh.ac.cn/), and NSII (http://www.nsii.org.cn/). Descriptions were based on morphological observations and measurements of living plants and herbarium specimens. Voucher specimens were deposited in the herbarium of the South China Botanical Garden (IBSC).

### Taxonomic treatment

#### Mussaenda saxatilis

Taxon classificationPlantaeGentianalesRubiaceae

T.T.Duan & J.Li sp. nov.

22F02B1D-DBE3-52D9-9089-B2E51F94484A

urn:lsid:ipni.org:names:77412364-1

Figs 1, 2

##### Type.

China • Guangxi: Chongzuo City, Longzhou County, Zhu Bu Township, in forest, on limestone, 22°29'11.76"N, 106°56'2.66"E, alt. 357 m, 1 May 2026 (fl), *T.T. Duan & J.Li DTT2026003* (Holotype: *DTT2026003* (a), IBSC; Isotypes: *DTT2026003* (b-d), IBSC).

**Figure 1.**
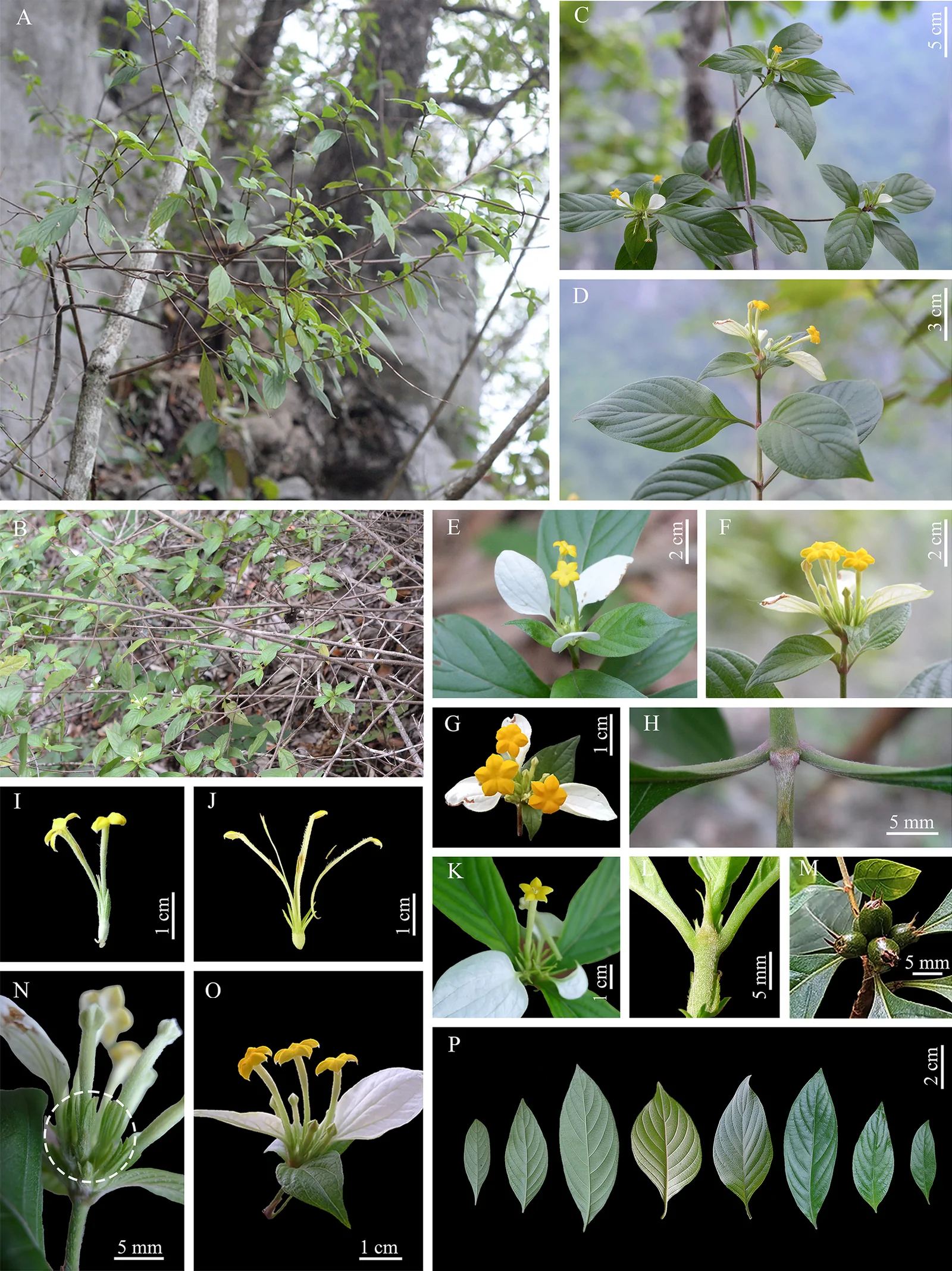
*Mussaenda saxatilis* T.T.Duan & J.Li. **A**. Habit; **B**. Plant; **C**. Inflorescence (long-styled flowers); **D**. Inflorescence (short-styled flowers); **E**. Long-styled flowers; **F**. Short-styled flowers; **G**. Flower, front view; **H**. Stipule; **I**. Longitudinally opened short-styled flower; **J**. Longitudinally opened long-styled flower; **K**. Long-styled flower, lateral view; **L**. Branch and stipule; **M**. Berry; **N**. Calyx lobes (white circle); **O**. Short-styled flower, lateral view; **P**. Leaves (first four: abaxial surface; last four: adaxial surface).

**Figure 2.**
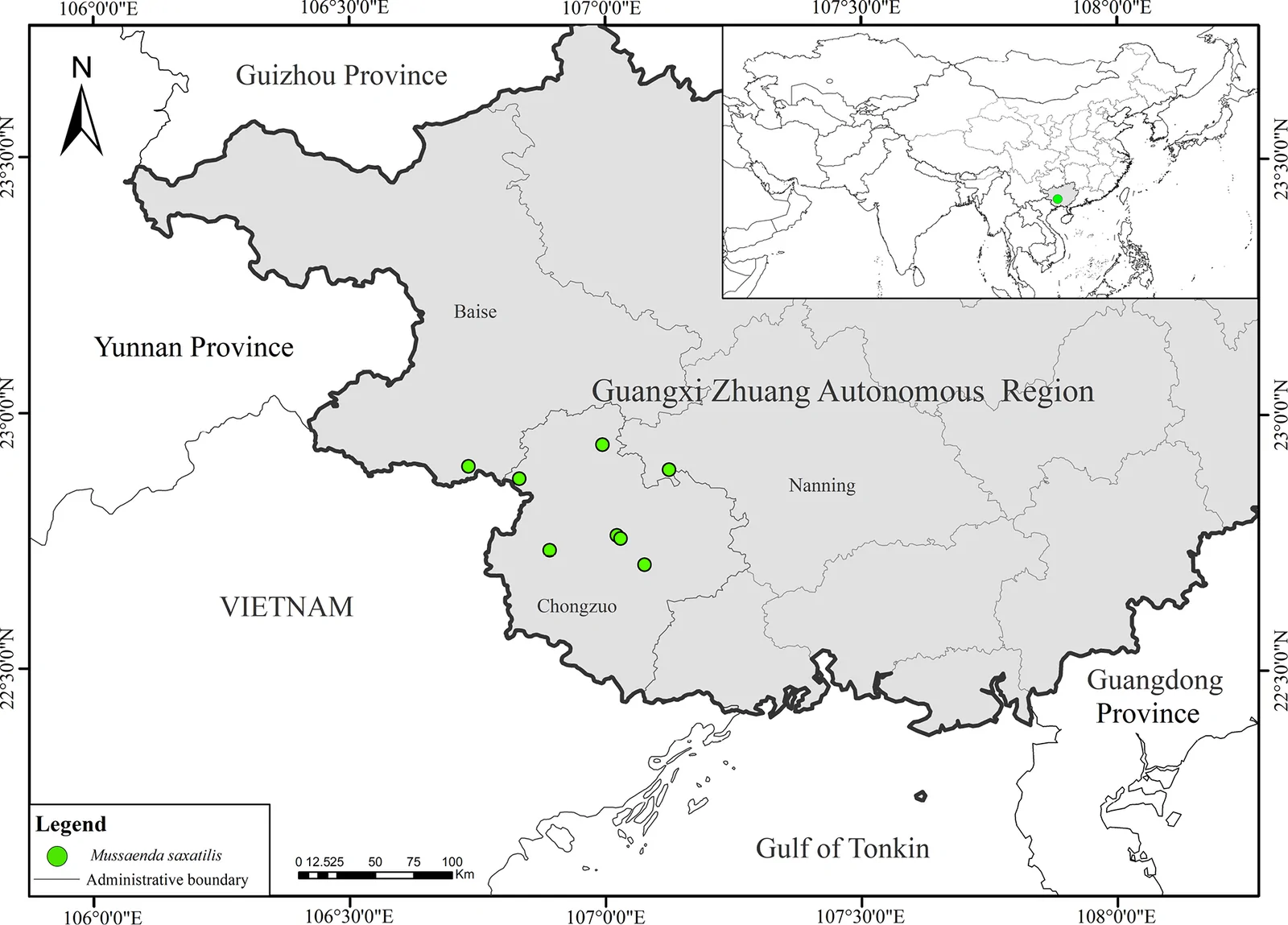
Distribution of *Mussaenda saxatilis* T.T.Duan & J.Li.

**Table 1.** Main morphological differences between *Mussaenda saxatilis* and *M. hainanensis*.

**Traits**	***M. saxatilis***	***M. hainanensis***
Petiole	0.3–1.5 cm	0.2–0.5 cm
Leaves	Ovate, lanceolate, elliptical, elliptic-oblong, 1.5–9.5 × 0.5–3.8 cm, abaxially sparsely velutinous to hirtellous, secondary veins 6–8 pairs	Oblong-elliptic, obovate, or lanceolate, 3–12 × 1.5–4 cm, abaxially densely villosulous or tomentulose, secondary veins 7–8 pairs
Stipules	Lanceolate to triangular, 3–8 mm long, shallowly bifid or cleft to the middle, lobes lanceolate to acuminate	Triangular to ovate, 3–7 mm long, entire or usually deeply 2-lobed, lobes lanceolate to linear, acuminate
Peduncle	0.2–0.6 cm	1–3 cm
Calyx lobes	Lanceolate to narrowly elliptic, 3–8 × 1.5–2 mm	Linear-lanceolate to narrowly elliptic, 6–8 × 1–2 mm
Calycophylls	Blade elliptic to ovate, 1.0–2.0 × 0.5–1.1 cm, stipe 3–5 mm long	Blade broadly elliptic to ovate, 1.5–4 × 1–3.5 cm, stipe 10–16 mm long
Corolla tube	20–33 mm long	20–25 mm long
Corolla lobes	Rotundate-ovate, 2–3 mm	Triangular-ovate, 3–5 mm
Fruits	Globose to subglobose, 4–6 × 5–8 mm, without lenticels	Ellipsoid to ellipsoid-oblong, 18–20 × 11–12 mm, densely lenticellate

##### Diagnosis.

*Mussaenda saxatilis* resembles *M. hainanensis* in its indumentum, leaf morphology, and yellow salverform corolla but differs in having stipules not deeply cleft, inflorescences subsessile, rotundate-ovate corolla lobes (vs. lobes triangular-ovate), smaller berries, and pericarp without lenticels.

##### Description.

Climbing shrubs, branches terete, ferruginous pubescent to strigillose. Leaves opposite, petiole 0.3–1.5 cm long, leaf blades ovate, lanceolate, elliptical, elliptic-oblong, 1.5–9.2 × 0.5–3.8 cm, papyraceous when dry, adaxially dark green, sparsely strigillose to hispid, abaxially strigillose or tomentulose, both surfaces with pubescence denser along principal veins, base cuneate or obtuse to rounded, apex acute to acuminate, secondary veins 6–8 pairs, tertiary venation visible and reticulate; stipules persistent, lanceolate to triangular, 3–8 mm long, densely pilosulous to villosulous outside, shallowly bifid or cleft to the middle, lobes lanceolate to acuminate. Inflorescences terminal, subcapitate to congested-cymose, 3–5 × 3–5 cm, densely pilosulous to villosulous; peduncles short, 2–6 mm long, bracts linear-lanceolate, 2–5 mm long. Flowers sessile or subsessile, calyx densely strigose to sericeous or pilose outside; hypanthium portion ellipsoid to turbinate, 1.5–3.0 mm long; lobes lanceolate to narrowly elliptic, 3–8 × 1.5–2 mm, densely strigillose to strigose outside, acute to acuminate, with one calyx lobe of one or a few flowers in each inflorescence expanded into calycophyll, blade broadly elliptic to ovate, 1.0–2.0 cm × 0.5–1.1 cm, adaxially moderately strigillose or sericeous, abaxially densely villosulous or tomentulose, base cuneate, obtuse, truncate, or cordulate, stipe 3–5 mm long, apex acute. Short-styled flowers: corolla yellow or orange-yellow, salverform, densely strigose outside; tube 20–27 mm long; densely clavate pubescent in throat; lobes rotundate-ovate, 2–3 mm long, adaxially densely yellow papillose, apex rounded; stamens 5, inserted at the middle-upper part of corolla tube; anthers linear, 3–4 mm long; style glabrate, stigma bifid, lobes equal and oblong. Long-styled flowers: flower morphology similar to short-styled flowers but calyx lobes shorter and corolla tube more slender, tube 24–33 mm long, stamens included in inflated lower part of corolla tube; anthers linear, 2–3 mm long; stigma exserted from the corolla tube, bifid, its lobes equal in size and oblong; style glabrate. Berry globose to subglobose, 4–6 × 5–8 mm, densely velutinous to hirtellous, without lenticels, calyx limb deciduous. Seeds numerous, minute, borne on axile placentas.

##### Chinese name.

shí shān yù yè jīn huā (石山玉叶金花).

##### Phenology.

Flowering from April to June; fruiting from June to November.

##### Distribution and habitat.

Currently known only from southern Guangxi, China. This species is confined to limestone karst habitats at elevations of 150–900 m a.s.l. (Fig. 2).

##### Etymology.

The species epithet “saxatilis” refers to its limestone habitat.

##### IUCN Red List category.

Based on current field investigations and herbarium records, *Mussaenda saxatilis* is known from approximately eight populations in southern Guangxi, China, all restricted to limestone karst habitats. These populations are scattered but occur within a relatively restricted geographic range. The species is confined to forested limestone hills at elevations of 150–900 m, ecosystems that are highly fragmented as a result of human disturbance. Although several populations have been documented, no quantitative data on population size, trends, or long-term dynamics are available. In addition, the potential occurrence of this species in adjacent regions of northern Vietnam remains uncertain. According to the IUCN Criteria (IUCN 2022), the available information is insufficient to make an assessment of extinction risk. Therefore, the species is currently assessed as Data Deficient (DD). However, given its strict habitat specificity and restricted known distribution, further field surveys are strongly recommended, as additional data may eventually justify its placement in a threatened category.

##### Additional specimen examined

**(paratypes)**. China • Guangxi Province: Chongzuo City, Longzhou County, Zhu Bu Township, 1 May 2026, *T.T.Duan & J.LI DTT2026004, DTT2026005, DTT2026006* (IBSC); • ibid., 15 July 2022, *S.Y.Nong, S.Y.Nong & X.Y.Huang ZYB02825* (PE), S.Q. Chen 13824 (IBK, HITBC); • ibid, 20 October 2010, *W.H.Wu W0213* (IBK); • ibid., 11 October 2008, *Guangxi Planting Joint Team 3704* (IBK); • ibid., 11 May 1980, *Nonggang Survey Team 12152* (IBK); • ibid., 25 September 1979, *Nonggang Survey Team 10423* (IBK); • Chongzuo City, Jiangzhou District, Zuozhou Town, 19 July 2021, *S.Y.Nong, S.Y.Nong & X.Y.Huang ZYB00858* (PE, IBK); • Chongzuo City, Fusui County, Changping Town, 24 May 2016, *D.X.Nong, Z.H.Nong & L.L.Mo 451421160524026LY* (GXMG); • Chongzuo City, Daxin County, Xialei Autonomous Regional Nature Reserve, 20 May 2008, *Guangxi Planting Joint Team 0164* (IBK); • Nanning City, Long’an County, Pingshan Township, 29 May 2006, *N.H.Tan, Y.Yan, M.F.Hou, Q.G.Shen, W.B.Xu & Y.Huang 529007* (PE, IBK); • Nanning City, Long’an County, Longhushan Nature Reserve, 11 May 2008, *Guangxi Planting Joint Team 0164* (IBK); • ibid., 13 May 2008, *Guangxi Planting Joint Team 0310* (IBK); • ibid., 9 July 91, *C.C.Huang 00986* (GXMI); • ibid., 1 September 2011, *J.C.Yang, R.C.Hu & J.Liu LH0491* (IBK); • ibid., 6 September 2011, *J.C.Yang, R.C.Hu & J.Liu LH0742* (IBK); • ibid., 21 November 2011, *J.C.Yang & Y.B.Liao LH1678* (IBK); • ibid., 4 November 2011, *J.C.Yang & Y.B.Liao LH0938* (IBK); • Nanning City, Long’an County, Dujie Town, Longge Village, 27 June 2014, *Long’an General Survey Team 450123140627048LY* (IBK, GXMG); • Jingxi City, Renzhuang Township, Bangliang Gibbon National Nature Reserve, 26 September 2019, *P. Yang, J.L.Li & Y.L.Su BL019* (IBK); • ibid., 29 September 2007, *W.B.Xu & Y.Y.Liang B0076* (IBK); • ibid., 9 August 2020, *P. Yang, S.Y. Nong, S.Y.Nong BL742* (IBK).

## Discussion

*Mussaenda saxatilis* is similar to *M. hainanensis* in its climbing habit, leaf shape variation, and yellow to orange-yellow corolla. However, it can be clearly distinguished from the latter by a combination of stable morphological characters, including stipules that are only shallowly to moderately bifid rather than deeply divided, subsessile inflorescences, rotundate-ovate corolla lobes, and fruits lacking lenticels. In addition, differences in calycophyll morphology and stipe length further support its separation from *M. hainanensis*. The latter typically exhibits larger calycophyll blades with longer stipes and distinctly lenticellate fruits (Chen and Taylor 2011). These diagnostic characters are consistent across all examined populations of *M. saxatilis*, suggesting that it represents a stable and independently evolving species within the genus *Mussaenda*. Previous herbarium misidentifications (e.g., PE02380316, PE02380317, IBK00454935) further indicate that this species has long been overlooked within *M. hainanensis*, highlighting the taxonomic complexity of the genus in southern China. The discovery of *M. saxatilis* contributes to a better understanding of species diversity within *Mussaenda* in China, particularly in karst ecosystems. Together with recent taxonomic advances in the genus (Duan et al. 2016, 2019), this study highlights the ongoing underestimation of species richness in southern China and emphasizes the importance of continued field exploration and herbarium revision.

## Supplementary Material

XML Treatment for Mussaenda saxatilis

## Additional information

### Artificial Intelligence (AI) use

The authors accept full responsibility for the content of the manuscript, including the disclosure of any use of AI.

No AI tools were used in the preparation of this manuscript.

### Author ORCIDs

J. Li https://orcid.org/0000-0001-5034-511X

T.-T. Duan https://orcid.org/0000-0003-2140-9967

### Data availability

All of the data that support the findings of this study are available in the main text.
